# Incidence of and risk factors for postoperative nausea and vomiting at a Japanese Cancer Center: first large-scale study in Japan

**DOI:** 10.1007/s00540-012-1468-5

**Published:** 2012-08-28

**Authors:** Ryozo Morino, Makoto Ozaki, Osamu Nagata, Miyuki Yokota

**Affiliations:** 1 Department of Anesthesiology, The Cancer Institute Hospital of Japanese Foundation for Cancer Research, 3-8-31, Ariake, Koto, Tokyo, 135-8550 Japan; 2 Department of Anesthesiology, Tokyo Women’s Medical University, 8-1, Kawadacho, Shinjyuku, Tokyo, 162-8666 Japan

**Keywords:** Postoperative nausea and vomiting, PONV, Prospective study, Risk factors, Japan

## Abstract

**Purpose:**

The first purpose of this study was to determine the incidence of postoperative nausea and/or vomiting (PONV) 0–48 h after anesthesia at a Japanese cancer center. The second purpose of this study was to collect information on PONV risk factors, independently, in the categories of patient-related, anesthesia-related, and surgery-related factors.

**Methods:**

The frequency of nausea and vomiting was prospectively investigated from 0 to 48 h after anesthesia in 1645 patients (11–94 years of age) at a single medical institution. The occurrence of nausea and vomiting and the use of antiemetics were recorded up to 48 h after anesthesia. Patient-related, anesthesia-related, and surgery-related factors were also recorded and submitted to multiple logistic regression analysis to determine the relationship of these factors to nausea and vomiting.

**Results:**

The incidences of nausea and vomiting from 0 to 24 h after anesthesia were 40 and 22 %, respectively. The incidences 24–48 h after anesthesia were 10 and 3 %, respectively. Female sex, previous history of PONV, prolonged anesthesia, and remifentanil use during surgery were identified as risk factors for both nausea and vomiting. The use of a volatile anesthetic, use of fentanyl during surgery, postoperative use of opioids, nonsmoking status, and drinking alcohol on 4 or fewer days per week were identified as risk factors for nausea alone.

**Conclusion:**

The incidence of and risk factors for PONV at a Japanese cancer center according to this study are comparable to those reported elsewhere.

## Introduction

Postoperative nausea and/or vomiting (PONV) is a significant postoperative complication that has been repeatedly investigated in surveys of incidence [1–4]. Many studies have sought to determine risk factors for PONV [1, 3, 5, 6]. Numerous antiemetics have been developed [7–11] and are used for PONV as recommended in various guidelines [12–14]. However, antiemetics such as 5-HT3 receptor antagonists, dexamethasone, and neurokinin-1 (NK1) receptor antagonists are still unapproved for use in Japan. This leaves metoclopramide as the only therapeutic option and deprives Japanese physicians of effective therapies for PONV. The unavailability of new antiemetics is partially attributable to a lack of large-scale studies to investigate the incidence of PONV in this country. Conclusive evidence that PONV is a significant concern in Japan with an incidence essentially no different from that in other countries would substantiate the approval of the new antiemetics, a class of agents widely used elsewhere, for use in Japan.

The first purpose of this study was to determine the incidence of PONV 0–48 h after anesthesia at a Japanese cancer center. The second purpose of this study was to collect information on PONV risk factors, independently, in the categories of patient-related, anesthesia-related, and surgery-related factors.

## Subjects, materials, and methods

This study was designed as a prospective cohort study to analyze the incidence of PONV and risk factors for PONV. Following internal ethics committee approval, the study was conducted at a single center from June 15 to September 30, 2010.

### Subjects

The subjects of the study were patients with an American Society of Anesthesiologists physical status (ASA PS) of I to III who underwent elective surgery with general or local anesthesia. The following patients were excluded: pregnant women, patients requiring intensive care unit treatment, patients receiving or scheduled to receive a mild or greater emetogenic anticancer drug from 6 days before study entry to 48 h after anesthesia, patients with a symptomatic brain metastasis, patients unable to communicate for a mental reason, and patients for whom any of the following antiemetics were used 48 or fewer hours before the start of anesthesia or during anesthesia: a 5-HT3 receptor antagonist, dexamethasone, phenothiazine drug product, butyrophenone drug product, benzamide drug product, dopamine receptor antagonist, antihistamine, or a NK1 receptor antagonist. Patients for whom none of the exclusion criteria applied and who provided informed consent in writing were included in the study.

### Anesthesia and postoperatively used drugs

No preanesthetic premedication drug was administered. Propofol was administered to induce anesthesia; rocuronium or vecuronium was administered as a muscle relaxant; remifentanil, fentanyl, or morphine hydrochloride was administered as an opioid; sevoflurane, propofol, or nitrous oxide was administered for maintenance of anesthesia; and sugammadex sodium or neostigmine was administered to reverse the muscle relaxants. The attending anesthesiologist selected the particular drugs as normal. The prophylactic administration of antiemetics was not allowed except for postoperative rescue. Metoclopramide, which is approved in Japan for PONV, was used as the antiemetic.

### Collecting patient data

The attending anesthesiologist recorded patient characteristics on the day before surgery. During anesthesia, the anesthesiologist recorded the disease name, surgery type, anesthesia used, type and dose of any anesthesia-related drugs used, and duration of anesthesia. Following anesthesia, a ward nurse recorded the antiemetic used.

### Evaluating nausea and vomiting

We determined that evaluating PONV until 48 h after anesthesia could provide data that would be useful in the future, because NK1 receptor antagonists, the 5-HT3 receptor antagonist palonosetron, and other long-acting drugs have recently been used for treating PONV. PONV was therefore evaluated from 0 to 2, 2 to 24, and 24 to 48 h after anesthesia. PONV was evaluated by an anesthesiologist in the operating room and a nurse in the wards. The evaluator recorded whether nausea was present, and if so, the severity and the number of vomiting or retching episodes. Nausea was defined as a subjective sensation of the desire to vomit by the patient without actual movement of the expelling muscles.

### Statistical analysis

The frequencies and percentages of the patient characteristics were calculated in each category, as follows. The median age and age range (minimum–maximum) were calculated along with the mean body weight and standard deviation. The mean opioid dose was calculated along with the standard deviation. For the duration of anesthesia, the lower quartile, median, and upper quartile were calculated. Next, the frequencies and percentages of subjects with nausea, vomiting, and PONV were calculated individually for each surgery type and risk factor at 0–2 h, 2–24 h, 24–48 h, 0–24 h overall, and 0–48 h overall after anesthesia. Logistic univariate regression analysis 0–2 h, 2–24 h, 24–48 h, 0–24 h overall, and 0–48 h overall after anesthesia was conducted to investigate the relationship of the risk factors to nausea, vomiting, and PONV frequency. The regression coefficients, standard errors, odds ratios (ORs), and 95 % confidence intervals (CIs) were calculated along with Wald *P* values. To avoid the problems of multicollinearity between individual risk factors, the significance of correlations was tested using Spearman’s correlation coefficient, and *r* values of ≥0.6 were defined as the presence of association. The 13 factors that had no multicollinearity issues and that were considered relevant to PONV were selected from the factors that univariate analysis suggested as being related and were submitted to logistic multivariate regression analysis by the forced-entry method. Adjusted ORs, 95 % CIs, and Wald *P* values were calculated. Seven of these 13 factors (sex, history of PONV, nonsmoking status, volatile anesthesia vs. total intravenous anesthesia [TIVA], nitrous oxide, reversal of muscle relaxation, and postoperative opioids) were dichotomous. Because the 13 factors did not include local anesthesia, multivariate analysis was performed after excluding the cases of local anesthesia. Remifentanil and fentanyl doses were analyzed as continuous variables, with adjusted ORs calculated every 1000 μg for remifentanil and every 100 μg for fentanyl. The category variables for the remaining 4 factors were as follows. For body mass index (BMI), ≥25 kg/m^2^ and <25 kg/m^2^, for duration of anesthesia, >1 h and ≤1 h, for alcohol drinking, on ≤4 days per week and >4 days per week, and for age, ≥50 years and <50 years. In all analyses a *P* value of less than 0.05 constituted statistical significance. SAS 9.2 (SAS Institute, Cary, NC, USA) was used to perform the statistical analyses.

## Results

All 1645 patients undergoing surgery who did not satisfy any of the exclusion criteria were included in the study. The breakdowns for the surgery types were as follows: gynecological surgery (GYN) 273 (17 %), breast surgery 399 (24 %), orthopedic surgery (ORT) 136 (8 %), respiratory surgery (RES) 100 (6 %), abdominal surgery (ABD) 599 (36 %), and otological surgery (OTO) 138 (8 %).

Patient baseline characteristics are shown in (Table 1). A total of 1608 patients (98 %) underwent general anesthesia, and 37 patients (2 %) underwent spinal anesthesia. The duration of anesthesia was 204 min (131–290 min) [median (quartile 1–quartile 3)]. Anesthesia type, drugs used, and postoperative analgesia are presented in detail in Table 2. The median fentanyl dose was 150 μg and the median remifentanil dose was 1500 μg. As postoperative analgesia, an epidural local anesthetic was used in 137 patients (8 %), an epidural opioid was used in 604 patients (37 %), fentanyl intravenous patient-controlled analgesia (IVPCA) was used in 83 patients (5 %), a nonsteroidal anti-inflammatory drug (NSAID) was used in 485 patients (29 %), and additional intravenous opioids (pentazocine or morphine) were used in 125 patients (8 %).

**Table 1:** Patient characteristics

Characteristic	
Sex	
Female *n* (%)	1051 (63.9)
Male *n* (%)	594 (36.1)
Age (years) median (range)	59 (11–94)
−29 *n* (%)	71 (4.3)
30–39 *n* (%)	154 (9.4)
40–49 *n* (%)	320 (19.5)
50–59 *n* (%)	303 (18.4)
60–69 *n* (%)	450 (27.4)
70– *n* (%)	347 (21.1)
Weight (kg) mean (SD)	57.4 (11.0)
BMI (kg/m^2^) mean (SD)	22.3 (3.4)
Nonsmoking status *n* (%)	1438 (87.4)
Drinking (on ≤4 days per week)	1265 (76.9)
History of PONV *n* (%)	135 (8.2)
History of motion sickness *n* (%)	454 (27.6)
ASA PS	
I *n* (%)	752 (45.7)
II *n* (%)	881 (53.6)
III *n* (%)	12 (0.7)

*ASA PS* American Society of Anesthesiologists physical status, *BMI* body mass index, *PONV* postoperative nausea and/or vomiting

**Table 2:** Perioperative drugs

Drug	*n* (%)	Median ± SD
General anesthesia (*n* = 1608)		
Induction		
Propofol	1608 (100)	
Anesthetic maintenance and reversal		
TIVA (propofol)	294 (18.3)	
Volatile anesthetic	1314 (81.7)	
With nitrous oxide	212 (13.2)	
Without nitrous oxide	1102 (68.5)	
With epidural anesthesia	743 (46.2)	
Muscle relaxant use	1563 (97.2)	
Muscle relaxants reversal use	1128 (70.1)	
Neostigmine	172 (15.2)	
Sugammadex	956 (84.8)	
Fentanyl (μg)	1482 (90.1)	150.0 ± 166.8
Remifentanil (μg)	1241 (75.4)	1500.0 ± 1867.1
Morphine (mg)	12 (0.73)	3.0 ± 2.2
48-h Postoperative analgesics		
Epidural local anesthetic	137 (8.3)	
Opioids^a^	709 (43.1)	
NSAIDs	485 (29.5)	

*TIVA* total intravenous anesthesia, *NSAIDs* nonsteroidal anti-inflammatory drugs

^a^Opioids include epidural opioids, fentanyl intravenous patient-controlled analgesia, and additional intravenous opioids (morphine, pentazocine)

The incidences of nausea, vomiting, and PONV and the usage of antiemetics are shown in Table 3 according to sex, type of anesthesia, and the type of surgery. The incidences of nausea and vomiting from 0 to 24 h after anesthesia were 40 and 22 %, respectively. Twenty-four percent of the patients used an antiemetic. The incidences of nausea and vomiting from 24 to 48 h after anesthesia were 10 and 3 %, respectively. The incidences of nausea and vomiting over the entire period studied were higher in the female patients. The incidences of nausea and vomiting after local anesthesia tended to be lower than those after general anesthesia, although there were far fewer cases of local anesthesia as compared with general anesthesia. The incidence of PONV from 0 to 48 h after anesthesia was 52 % for GYN, 53 % for BRE, and 46 % for OTO. These figures were higher than the 32 % for ORT, 30 % for RES, and 35 % for ABD. The incidence of PONV from 24 to 48 h after anesthesia was 19 % for GYN, 13 % for RES, and 12 % for ABD, values which were higher than the 3.3 % for BRE, 5.9 % for ORT, and 3.5 % for OTO.

**Table 3:** Frequencies of PONV and antiemetic administration in the various periods

Outcome	Sex		Type of anesthesia		Type of surgery						Total (*n* = 1645)
	Male (*n* = 594)	Female (*n* = 1051)	GA (*n* = 1608)	RA (*n* = 37)	GYN (*n* = 273)	BRE (*n* = 399)	ORT (*n* = 136)	OTO (*n* = 138)	RES (*n* = 100)	ABD (*n *= 599)	
0–2 h											
Nausea	10.6	30.1	23.5	8.1	32.6	29.1	14.0	26.2	12.0	17.7	23.0
Vomiting	4.4	12.2	9.6	0.0	11.7	10.8	5.1	14.2	3.0	8.2	9.4
PONV	10.6	30.3	23.7	8.1	32.6	29.1	14.0	27.0	12.0	17.9	23.2
Antiemetics	4.5	15.7	12.0	0.0	19.4	14.5	5.1	17.0	1.0	8.2	11.7
2–24 h											
Nausea	16.7	40.1	32.4	5.4	35.5	42.1	25.0	31.9	24.0	25.4	31.6
Vomiting	8.4	21.8	17.3	5.4	15.8	23.3	12.5	18.4	15.0	14.2	17.0
PONV	16.7	40.2	32.5	5.4	35.5	42.1	25.0	31.9	24.0	25.7	31.7
Antiemetics	5.4	22.6	16.8	2.7	20.1	24.6	11.8	17.7	8.0	11.4	16.4
24–48 h											
Nausea	6.1	11.8	9.9	2.7	18.7	3.3	5.9	3.5	13.0	11.7	9.7
Vomiting	1.5	3.5	2.8	2.7	6.6	0.8	0.7	1.4	3.0	3.2	2.8
PONV	6.1	12.1	10.1	2.7	19.4	3.3	5.9	3.5	13.0	11.9	9.9
Antiemetics	2.2	5.7	4.6	0.0	8.8	0.8	3.7	2.8	3.0	5.7	4.4
0–24 overall											
Nausea	21.7	51.0	41.4	10.8	48.4	52.9	31.6	45.4	28.0	31.2	40.4
Vomiting	10.9	28.4	22.6	5.4	23.1	28.3	16.9	26.2	19.0	18.0	22.1
PONV	21.7	51.3	41.6	10.8	48.4	52.9	31.6	45.4	28.0	31.7	40.6
Antiemetics	9.3	32.0	24.4	2.7	31.9	33.8	16.9	28.4	8.0	16.4	23.8
0–48 overall											
Nausea	24.1	52.6	43.3	10.8	52.0	52.9	31.6	46.1	30.0	34.2	42.3
Vomiting	11.6	29.1	23.3	5.4	24.2	28.6	16.9	27.0	20.0	19.0	22.8
PONV	24.1	53.0	43.6	10.8	52.4	52.9	31.6	46.1	30.0	34.7	42.6
Antiemetics	10.6	33.5	25.9	2.7	34.4	34.1	19.1	29.1	10.0	18.0	25.2

*GA* general anesthesia, *RA* regional anesthesia, *GYN* gynecological surgery, *BRE* breast surgery, *ORT* orthopedic surgery, *RES* respiratory surgery, *ABD* abdominal surgery, *OTO* otological surgery, *PONV* postoperative nausea and/or vomiting

The adjusted ORs and CIs of the risk factors for nausea and vomiting 0–48 h after anesthesia are shown in Table 4. Patient-related risk factors for both nausea and vomiting were female sex and history of PONV. Female sex was a particularly strong risk factor, with adjusted ORs (CIs) of 4.1 (3.00–5.62) and 3.65 (2.52–5.30), respectively. Nonsmoking status and drinking alcohol on 4 or fewer days per week were identified as risk factors for nausea alone. Anesthesia-related risk factors for both nausea and vomiting were duration of anesthesia exceeding 1 h and the intraoperative use of remifentanil. The use of a volatile anesthetic (versus propofol), the intraoperative use of fentanyl, and the postoperative use of opioids were identified as risk factors for nausea alone. The results did not show age, BMI, nitrous oxide use, or muscle relaxant antagonists to be correlated with nausea or vomiting.

**Table 4:** Adjusted odds ratios of risk factors for nausea and vomiting within the 0–48 h overall period

Factor	Odds ratio	95 % CI		*P* value
		Lower	Upper	
Nausea (0–48 h)				
Sex (female)	4.10	3.00	5.62	<0.0001
Age (≥50 years)	0.87	0.65	1.16	0.3407
History of PONV	2.04	1.29	3.23	0.0022
Nonsmoking status	1.56	1.05	2.33	0.0256
BMI (kg/m^2^) (≥25)	1.08	0.79	1.48	0.6394
Drinking (on ≤4 days per week)	1.39	1.02	1.92	0.0374
Duration of anesthesia (>1 h)	2.97	1.87	4.71	<0.0001
Volatile anesthesia vs. TIVA	1.61	1.14	2.22	0.0066
Nitrous oxide	1.19	0.74	1.92	0.4641
Remifentanil (per 1000 μg)	1.23	1.11	1.36	<0.0001
Fentanyl (per 100 μg)	1.17	1.04	1.33	0.0103
Reversal of muscle relaxation	1.29	0.97	1.73	0.0849
Postoperative opioids	1.38	1.03	1.83	0.0284
Vomiting (0–48 h)				
Sex (female)	3.65	2.52	5.30	<0.0001
Age (≥50 years)	1.48	1.06	2.06	0.0214
History of PONV	1.87	1.20	2.91	0.0056
Nonsmoking status	1.27	0.79	2.00	0.3177
BMI (kg/m^2^) (≥25)	0.81	0.57	1.16	0.2531
Drinking (on ≤4 days per week)	1.39	0.95	2.04	0.0905
Duration of anesthesia (>1 h)	1.77	1.02	3.06	0.0426
Volatile anesthesia vs. TIVA	1.16	0.81	1.69	0.4180
Nitrous oxide	1.02	0.59	1.75	0.9400
Remifentanil (per 1000 μg)	1.28	1.16	1.41	<0.0001
Fentanyl (per 100 μg)	1.09	0.96	1.23	0.1917
Reversal of muscle relaxation	1.25	0.89	1.74	0.1921
Postoperative opioids	1.21	0.89	1.65	0.2286

*CI* confidence interval, *PONV* postoperative nausea and/or vomiting, *BMI* body mass index, *TIVA* total intravenous anesthesia

**Fig. 1:**
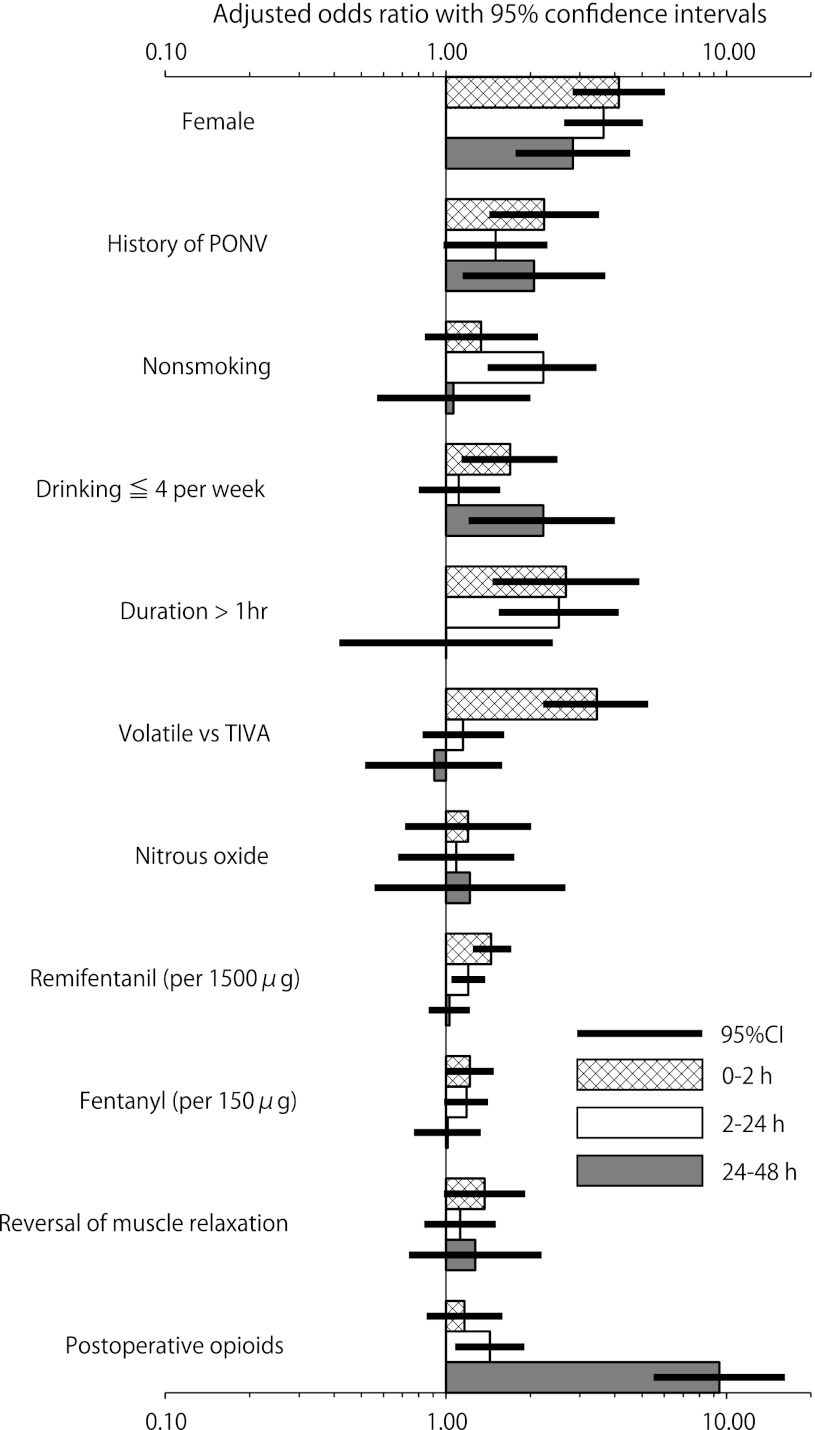
Strengths of PONV risk factors at 0–2, 2–24, and 24–48 h after anesthesia. Note that the *x* axis is log-scale. *PONV* postoperative nausea and/or vomiting, *CI* confidence interval, *drinking ≤4 per week* drinking on ≤4 days per week, *duration* duration of anesthesia, *volatile* volatile anesthetic, *TIVA* total intravenous anesthesia

The strengths of the PONV risk factors at 0–2 h, 2–24 h, and 24–48 h after anesthesia are shown in Fig. 1. The use of a volatile anesthesic was highly significant, with an adjusted OR (CI) of 3.45 (2.22–5.26) from 0 to 2 h after anesthesia, but this factor was unrelated to PONV 2–24 h and 24–48 h after anesthesia (*P* = 0.40, *P* = 0.73). The use of remifentanil (in 1500 μg increments) was significant, with an adjusted OR (CI) of 1.65 (1.34–2.03) 0–2 h after anesthesia, but its significance decreased to an adjusted OR (CI) of 1.27 (1.06–1.53) 2–24 h after anesthesia and it was insignificant 24–48 h after anesthesia (*P* = 0.79). The use of fentanyl (in 150 μg increments) was significant, with adjusted ORs (CIs) of 1.28 (1.00–1.63) 0–2 h after anesthesia and 1.23 (0.99–1.54) 2–24 h after anesthesia, but it was insignificant 24–48 h after anesthesia (*P* = 0.93). The postoperative use of opioids was insignificant 0–2 h after anesthesia (*P* = 0.33), but was highly significant, with adjusted ORs (CIs) of 1.43 (1.08–1.90) 2–24 h after anesthesia and 9.42 (5.50–16.16) 24–48 h after anesthesia. The effects of female sex and history of PONV, which were risk factors for both nausea and vomiting, were consistent across the time periods studied.

## Discussion

This large-scale study, which was conducted to identify PONV incidence and risk factors, is the first such study to demonstrate the high incidence of PONV in Japan. The study also showed delayed incidencesm from 24 to 48 h after anesthesiam to be relatively high, at 10 % for nausea and 3 % for vomiting.

When interpreting the incidences of PONV in the present study, it must be remembered that the median duration of anesthesia was 204 min, which was longer than that in other studies [1–4]. Furthermore, comparing incidences of PONV among studies may not be feasible because of the difficulty involved with matching patient-, anesthesia-, and surgery-related factors. We believe that another study of PONV incidences in Japan, with matched risk factors is necessary.

PONV incidence is known to be high for certain types of surgeries [2, 15, 16]. The present study showed a high incidence of PONV for GYN, BRE, and OTO. It should be noted that most or all of the patients undergoing these types of surgeries were female. Some investigators claim that the relationship between surgery type and PONV is open to interpretation because patient characteristics and anesthesia-related factors are at play [1, 5, 17, 18]; accordingly, the effect of surgery type was excluded from multivariate regression analysis in the present study.

In the present study, female sex and a history of PONV were identified as risk factors for both nausea and vomiting. Female sex and a history of PONV have previously been identified as important PONV risk factors [2–4, 18]. We also showed that nonsmoking status and drinking alcohol on 4 or fewer days per week were risk factors for nausea alone. Nonsmoking status has previously been shown to increase PONV incidence [19, 20]. Sweeney [20] states that higher levels of drinking may suppress PONV. Our findings validate this prediction.

ASA PS and BMI are reported to affect PONV [2, 3, 21], but these factors were not found to be related to PONV in our study.

Longer durations of anesthesia and the use of volatile anesthetics have been previously reported as risk factors for PONV [22, 23]. The present study showed the risk of PONV to increase with increasing opioid doses during surgery; increasing the remifentanil dose by 1000 μg increased the adjusted OR for nausea by 1.23-fold, while increasing the fentanyl dose by 100 μg increased the OR by 1.17-fold within the 0–48 h overall period. Postoperative opioid use is believed to increase PONV in a dose-dependent manner [24] and was shown in our study to be a risk factor for nausea. The use of nitrous oxide is also thought to be a risk factor for PONV [25–27], but this factor did not have an impact in our study.

The use of 2.5 mg or more neostigmine is a reported risk factor for PONV [28], but our study revealed no relationship between muscle relaxant antagonist use and PONV, possibly because sugammadex was frequently used but neostigmine was not. Although further research is required, because previous univariate analysis of the effects of sugammadex on PONV showed no relationship, it seems that sugammadex may not affect PONV.

Apfel et al. [22] state that volatile anesthetics are a strong risk factor for PONV for up to 2 h after surgery. Stadler et al. [4] disagree with this conclusion, attributing it to the inclusion of a high proportion of children and imbalances in surgery types. Although the present study had a higher proportion of female patients (64 vs. 53 %) than the study of Stadler et al. [4], the proportion of child patients was comparable to the proportion in their study, and our study had no surgery type imbalances such as those in the study of Apfel et al. [22]. We found that the use of volatile anesthetics was a risk factor for nausea, with an adjusted OR of 3.45, only 0–2 h after anesthesia. This strengthens the claim of Apfel et al. [22] and indicates that the impact of volatile anesthetics is very substantial during the early period 0–2 h after anesthesia.

With regard to perioperatively used opioids, our study evaluated the intensity of the effects of not only according to dose but also according to time after anesthesia. Postoperative opioid use, which produces PONV 2 h after anesthesia and beyond, has been shown to be a significant factor for delayed PONV 24 h and beyond. In the present study, the incidence of PONV was relatively high for GYN, RES, and ABD 24–48 h after anesthesia; postoperative epidural opioids and fentanyl IVPCA were used more frequently after these types of surgery than the others. We found that intraoperative remifentanil use had a large impact on PONV 0–2 h after anesthesia. The effects of its use were diminished 2–24 h after anesthesia and were not observed 24 h after anesthesia and beyond. Intraoperative fentanyl use had a comparable impact 0–24 h after anesthesia, but no effects were noted 24 h after anesthesia and beyond. Although our study did not closely compare the significance of the effects of remifentanil and fentanyl by excluding other factors, as in the study by Rama-Maceiras et al. [29], the adjusted ORs show that the times of impact and intensities of PONV differed according to the particular opioid used.

Problematically, the hospital stay of ambulatory surgery patients may be prolonged by PONV attributable to opioid use [13–15]. The findings of the present study suggest that reducing opioid dosage and, particularly, reducing intraoperative fentanyl and postoperative opioid use could be effective for preventing prolonged hospital stays and delayed PONV.

In overall consideration of the results and given the difficulty of controlling patient-related factors in the clinic, controlling anesthesia-related factors appears to be an effective way of reducing the incidence of PONV. It is important to avoid using volatile anesthetics, which are a strong risk factor for nausea during the 2-h period following anesthesia, and to also reduce the use of opioids, which are a risk factor for delayed PONV. However, reducing opioid use has been shown to lead to an increase in anesthetic use [30, 31]; so, using propofol, whose dose Apfel et al. [22] claim is not proportional to PONV, and using local anesthesia and NSAIDs as proposed by Shirakami et al. [32] appear to be viable options for reducing opioid use as much as possible.

No large-scale studies of PONV incidence to support the use of new antiemetics and no evaluations of the efficacy of antiemetics used per consensus guidelines for PONV have been conducted at any Japanese Cancer Center or at any other institution in Japan. Further investigation will be required to determine how PONV incidence, as defined in the present research, can be decreased through prophylaxis with antiemetics. A risk score must be constructed on the basis of the data obtained by this study and evaluated for effectiveness.
